# Impact of Dietary Shrimp Waste on Physical Properties, Chemical Composition, Amino Acid Profile, and Antioxidant Levels of Breast Meat

**DOI:** 10.3390/vetsci12121130

**Published:** 2025-11-27

**Authors:** Esin Ebru Onbaşılar, Umut Can Gündoğar, Hilal Çapar Akyüz, Yeliz Kaya Kartal, Sakine Yalçın, Emirhan Nemutlu, Tuba Reçber, Mustafa Feyzullah Akyüz, Duru Onbaşılar, Banu Yüceer Özkul, Necmettin Ünal, Ceyhan Özbeyaz

**Affiliations:** 1 Department of Animal Husbandry, Faculty of Veterinary Medicine, Ankara University, 06110 Ankara, Turkey; 2 Department of Biochemistry, Faculty of Veterinary Medicine, Ankara University, 06110 Ankara, Turkey; ylzkaya@ankara.edu.tr; 3 Animal Nutrition Science Association, 06420 Ankara, Turkey; 4 Department of Analytical Chemistry, Faculty of Pharmacy, Hacettepe University, 06100 Ankara, Turkey; 5 The Graduate School of Health Sciences, Ankara University, 06110 Ankara, Turkey; akyuzmf@ankara.edu.tr; 6 Department of Fisheries and Aquaculture, Faculty of Agriculture, Ankara University, 06120 Ankara, Turkey

**Keywords:** meat quality, amino acid profiles, antioxidant capacity, shrimp waste, broiler

## Abstract

Utilizing waste materials in broiler diets supports the principles of the circular economy and promotes environmental sustainability. However, it is essential to ensure that such practices do not compromise meat quality or composition. This experiment evaluated how incorporating shrimp processing waste into broiler diets influences breast meat physicochemical properties, nutrient composition, amino acid profiles, and antioxidant properties. A total of 315 Ross 308 male broiler chicks were allocated to diets containing varying inclusion levels of shrimp waste and reared for 42 days. At the end of the feeding period, seven birds from each treatment group were selected for slaughter, and a total of 35 breast muscle samples were collected for laboratory analyses. Parameters including pH, color, water-holding capacity, cooking loss, nutrient content, amino acid composition, and antioxidant indices were determined. Dietary shrimp waste did not influence pH_15_ but increased pH_24_ values. Lightness and redness remained unaffected, whereas yellowness increased. Cooking loss, water-holding capacity, and nutrient composition were not significantly altered. Alanine and aspartic acid levels decreased in groups supplemented with shrimp waste, whereas glycine content increased in the group receiving 1% shrimp waste during the first 10 days. Antioxidant parameters and the oxidative stress index were also unchanged. These findings indicate that incorporating shrimp waste into broiler diets can enhance feed sustainability without compromising meat quality.

## Simple Summary

This study evaluated the effects of including shrimp-processing waste in broiler diets on breast meat quality and composition. A total of 315 Ross-308 male broilers were fed diets containing 0%, 1%, or 2% shrimp waste during different growth phases for 42 days. Breast meat samples were analyzed for pH, color, water-holding capacity, cooking loss, nutrient content, amino acid composition, and antioxidant capacity. Inclusion of shrimp waste did not affect meat pH_15_, but increased meat pH_24_ (*p* < 0.001). Neither lightness nor redness values showed any noticeable variation among the treatments, whereas the yellowness parameter was markedly elevated (*p* < 0.001). Additionally, cooking loss, water-holding capacity, and the nutrient composition of the meat were not significantly affected by the dietary treatments (*p* > 0.05). Amino acid and antioxidant profiles also showed no major changes, except for minor variations in alanine, aspartic acid, and glycine (*p* < 0.05). Overall, shrimp waste had no adverse effect on meat quality, indicating its potential as a sustainable ingredient in broiler diets.

## 1. Introduction

The global demand for poultry production has been rising significantly over recent decades. However, ensuring sustainability in the industry requires not only an emphasis on production and meat yield but also a focus on enhancing meat quality parameters [1,2]. Broiler meat quality is generally assessed in three main aspects: appearance (e.g., flesh color), physical properties (e.g., muscle pH, water-holding capacity, and texture), and chemical composition (e.g., crude protein and fat content) [3]. Animal-derived foods constitute a fundamental component of the human diet and have long played a vital role in meeting nutritional requirements [4,5]. Animal-based proteins play a vital role in human nutrition, supplying high-quality protein, essential amino acids, and critical micronutrients.

Pre-slaughter stressors and post-slaughter storage conditions are known to elevate levels of reactive oxygen species and reactive nitrogen species in muscle tissues. This oxidative stress impairs sensory properties, diminishes protein functionality, and depletes amino acids [6,7]. Notably, myofibrillar proteins, which constitute a significant portion of total muscle protein, are particularly susceptible to oxidative damage, resulting in structural and functional alterations that manifest as discoloration and flavor changes in meat [6,8]. To address these challenges, poultry companies are exploring novel strategies, such as dietary supplementation with natural antioxidants, to mitigate oxidative damage and improve meat quality [9]. Incorporating antioxidants into poultry diets can reduce protein oxidation, thereby enhancing post-slaughter meat quality. Natural antioxidants, in particular, are gaining popularity due to their perceived safety, health benefits, and consumer acceptability [10].

While amino acids are primarily recognized as essential components of protein synthesis, they also play key roles in various metabolic functions, including gene expression, hormonal regulation, oxidative defense, nutrient metabolism, and immune responses [11]. Functional amino acids such as arginine, cysteine, leucine, and tryptophan are particularly important in promoting optimal growth, immunity, and overall physiological function in broilers [12].

The evaluation of meat quality traits is fundamental for elucidating the nutritional, technological, and sensory characteristics of broiler meat. Physical traits including pH, water-holding capacity (WHC), cooking loss, and color are well known to influence the visual quality of meat, tenderness, juiciness, and overall acceptance by consumers [13,14]. The ultimate pH, measured 24 h postmortem, plays a critical role in determining WHC, texture, and color, and is therefore considered one of the key indicators of meat quality [15]. WHC, in turn, governs drip loss, cooking yield, and the ability of the meat to retain moisture during processing and storage [16]. The chemical constituents of broiler meat, such as moisture, crude protein, fat, and ash, together with its amino acid composition offer critical insights into its nutritional quality and protein value. These parameters are fundamental for evaluating feed efficiency, developing appropriate diet formulations, and ensuring accurate information for consumers [17]. Moreover, the assessment of antioxidant capacity and lipid/protein oxidation (e.g., TBARS, carbonyls) is vital, as these parameters strongly influence the oxidative stability, shelf life, and sensory quality of poultry meat [18]. Taken together, a comprehensive characterization of physical, chemical, and oxidative parameters is necessary to scientifically evaluate how dietary factors, genetic background, management practices, feed composition, processing methods, and environmental factors influence the overall quality of broiler meat and its acceptance by consumers [17,18,19].

Among these, feed quality and cost are particularly critical for sustaining growth in the poultry sector. Utilizing alternative feed resources, such as animal by-products, offers a sustainable solution that addresses both economic and environmental concerns. Shrimp waste (SW), in particular, stands out as a viable protein-rich alternative to traditional ingredients such as soybean meal, which is subject to price fluctuations [20]. Ojewola and Udom [21] reported that shrimp meal contains approximately 59% crude protein (CP), 4% ether extract (EE), 1% crude fibre (CF), and 18% crude ash (CA), on a dry matter (DM) basis. Similarly, other researchers [20,22,23] have indicated that shrimp meal typically consists, on a DM basis, of 44–48% CP, 4–11% EE, 4% CF, and 7–28% CA, depending on the processing methods and shrimp species. Additionally, shrimp-derived astaxanthin, a potent lipid-soluble carotenoid, exhibits exceptional antioxidant properties, surpassing those of other carotenoids, vitamin E, and vitamin C in mitigating oxidative stress [24,25].

Hardini and Djunaidi [26] reported that fermented SW meal can be included in broiler diets at up to 7.5% to improve meat texture and color (lightness, redness, and yellowness). The pH, water content, and ash content were similar to the control; fat content was lower, and protein content was higher than in the control [26]. Ingweye et al. [27] concluded that 0% (control) and 25% replacement of fish waste meal with SW meal were optimal for broiler performance. According to the results of Onbaşılar et al. [2], up to 2% SW can be added to the diet without adversely affecting broiler performance, carcass quality, villus and bone properties, and fatty acid composition of meat. The skin color was not significantly affected by the use of 4%, 8%, and 12% shrimp meal; however, the redness of thigh meat increased due to the astaxanthin content [28]. Noaman and Bandr [29] concluded that SW can be used in broiler diets at levels of 4% and 6% to improve physiological and microbial characteristics and indicators of fat oxidation in the meat, and to allow storage of chicken meat for 30 days without deterioration of its quality. Khempaka et al. [28] evaluated the use of shrimp meal in broiler diets and reported that inclusion levels of 4, 8, and 12% numerically reduced body weight gain, feed intake, and feed efficiency. Increasing levels of shrimp meal also decreased dry-matter digestibility, ash digestibility, and nitrogen retention, likely due to the indigestibility of chitin in shrimp meal. Therefore, inclusion levels at or below 2% are commonly considered safe and nutritionally acceptable for broilers. Based on these findings, we selected 1% and 2% as biologically relevant and practically applicable levels for this study. Shrimp processing waste has a variable nutrient composition, and its availability and cost-effectiveness in commercial feed mills generally support its use at low inclusion levels. Thus, incorporation of 1% and 2% SW represents realistic supplementation rates that could be adopted by the poultry industry without compromising diet formulation or increasing feed costs. Accordingly, the present research examines the incorporation of 1% and 2% SW into broiler diets and evaluates how these inclusion levels influence breast meat characteristics, including appearance, physicochemical traits, chemical composition, amino acid profile, and antioxidant capacity. By addressing the dual objectives of improving broiler meat quality and promoting environmental sustainability, this research contributes to advancing sustainable poultry production systems.

## 2. Materials and Methods

### 2.1. Animals, Housing, General Management, and Breast Meat Samples

A total of 315 Ross-308 male broiler chicks were fed diets containing different levels of shrimp waste (*Penaeus vannamei*) for a 42-day feeding period: control, 1% during the first 10 days, 1% throughout the trial, 2% during the first 10 days, and 2% throughout the trial (Table 1). Each group was subdivided into seven replicates, with nine chicks assigned to each replicate, resulting in a total of 35 pens. The pens (90 × 80 cm) were provided with bedding material. A lighting regimen of 23 h of light and 1 h of darkness was applied on the first day, after which it was modified to 18 h of light and 6 h of darkness from the second week through the end of the experiment. The temperature at the chick level was initially set to 33 ± 1 °C for the first three days and subsequently reduced by 3 °C per week until the birds were 21 days old. The broilers received a starter diet from day 0 to day 11, a grower diet from day 11 to day 25, and a finisher diet from day 25 to day 42. The SW, mainly composed of head portions, was used frozen; it was thawed at room temperature and then dried in a forced-air oven at 55 °C for approximately 10 h. After drying, the material was ground into a fine powder using a 1.0 mm sieve and incorporated into the experimental diets. The SW prepared in this manner contained 92.67% DM, 52.65% CP, 9.55% EE, and 18.49% CA. The free and total amino acid compositions of the SW are provided in Table 2, whereas Table 3 presents the ingredients and chemical composition of the diets. At 42 days of age, one broiler whose body weight was close to the group mean was selected from each replicate group. Therefore, seven broilers from each diet group were exsanguinated via the jugular vein, and subsequently, thirty-five breast muscle samples were collected [2,30]. All measurements were performed on the upper one-third of the left side of the breast muscle.

Animal experiment was approved by the Animal Care and Use Committee of Ankara University (2022-17-158).

### 2.2. Appearance and Physical Properties of Breast Meat

The pH of the breast muscle was recorded at 15 min and 24 h postmortem using a pH meter (Mettler Toledo, Columbus, OH, USA). Before measurements, the device was standardized with pH 4.00, 7.00, and 10.00 buffer solutions at room temperature in accordance with the manufacturer’s instructions. The electrode was inserted into the central region of the breast muscle to ensure proper contact with tissue fluids; the reading was taken once the measurement stabilized, which typically required about 15 s [30]. The colour measurements for lightness (L*), redness (a*), and yellowness (b*) were also obtained at 15 min and 24 h of slaughter, using a CR-400 colorimeter (Konica Minolta, Tokyo, Japan) [30,31,32]. Prior to analysis, the instrument was calibrated using the white calibration plate supplied by the manufacturer. Samples were positioned flat on a clean tray to create a smooth surface; the colorimeter was positioned perpendicular to the tissue while avoiding connective tissue, blood clots, and intramuscular fat. Care was taken to ensure complete contact with the meat surface and to prevent any external light interference [30]. Cooking loss was determined by recording the initial weight (W1) of each breast meat sample, placing the samples in heat-resistant plastic bags, and heating them in a water bath at 80 °C for 60 min. After cooking, the samples were reweighed (W2), and the cooking loss was calculated as [(W1 − W2)/W1] × 100 [33]. The water-holding capacity values for breast meat were determined by measuring the amount of released water using a modified method of Hashizawa et al. [34], which is comparable to the expressible juice technique reported by Qiao et al. [14]. The samples collected from the same anatomical location were placed into 15 mL centrifuge tubes lined with filter paper and centrifuged at 2500× *g* for 20 min at 4 °C. All WHC analyses were completed within 24 h post-sampling. WHC was calculated as 100 × ((weight before centrifugation − weight after centrifugation)/weight before centrifugation); a greater percentage of water released indicates lower WHC.

All measurements were performed in triplicate at room temperature, and the average of the three readings was used for statistical evaluation.

### 2.3. Nutrient Composition of Shrimp Waste, Diets, and Breast Meat Samples

Proximate composition analyses of SW and the diets were carried out following the procedures outlined by AOAC [35]. The metabolizable energy content of the diets was calculated using the equation proposed by Carpenter and Clegg, as cited by Yalçın et al. [36]. Breast meat samples were first freeze-dried using a lyophilizer and then ground to a fine powder prior to nutrient analysis. The free and total amino acid profiles of SW were assessed according to the methods described by Yıltırak et al. [37]. All measurements were conducted in triplicate to ensure accuracy and reproducibility.

### 2.4. Amino Acid Profiles of Breast Meat

A 100 mg portion of each chicken breast meat sample was weighed, and 1 mL of a methanol:water mixture (9:1, *v*/*v*) was added for amino acid extraction. Samples were homogenized in an ultrasonic bath on ice, then vortexed and centrifuged at 14,000 rpm for 10 min. The resulting supernatant was transferred into HPLC vials for analysis. Amino acid quantification was carried out using a validated LC-ESI-MS/MS method. Calibration solutions were prepared with amino acid standards at concentrations of 0.10, 0.20, 0.50, 1.00, 2.00, and 5.00 µg/mL, each containing the internal standard at 1.00 µg/mL. The LC-ESI-MS/MS system (Shimadzu, Japan) consisted of a Shimadzu LC-20AXR liquid chromatograph coupled to a Shimadzu 8030 MS/MS triple-quadrupole mass spectrometer (Shimadzu, Kyoto, Japan), operated using LabSolutions software (Version 5.72). Chromatographic separation was performed on a ZIC-HILIC column (Merck, Darmstadt, Germany; 100 × 4.6 mm, 5 µm) at 60 °C using a gradient of water with 0.1% formic acid (mobile phase A) and acetonitrile with 0.1% formic acid (mobile phase B), at a flow rate of 0.4 mL/min. The gradient program was: 0–0.01 min, 80% B; 0.01–1.00 min, 80% to 90% B; 1.00–6.00 min, 90% to 5% B; 6.00–8.00 min, 5% B; 8.00–10.00 min, 5% to 90% B; followed by a 2-min re-equilibration at initial conditions (total run time: 12 min). The injection volume was 10 µL. Amino acids were quantified in positive ionization mode using multiple reaction monitoring (MRM). The optimal MS parameters were set as follows. interface voltage, 4.5 kV; collision energy, −14 V; desolvation line temperature, 250 °C; heat block temperature, 400 °C; nebulizer and drying gas flow rates of 3 and 15 L/min, respectively. Precursor and product ions, quadrupole voltages, and collision energies were optimized by direct injection of amino acid standards and the internal standard (13C-methionine). The optimized MRM conditions for the targeted amino acids were those described by Onbaşılar et al. [18]. Calibration curves were generated using least-squares linear regression by plotting the peak area ratio (amino acid/internal standard, y) against concentration (x). Sample amino acid concentrations were calculated based on these calibration equations.

Total essential amino acids (EAA; lysine, leucine, isoleucine, valine, methionine, threonine, phenylalanine, histidine, tyrosine, arginine, and tryptophan) and nonessential amino acids (NEAA; aspartic acid, glutamic acid, serine, glutamine, alanine, glycine, asparagine, cysteine, proline, hydroxyproline) were determined. The ratios of EAA to total amino acids and the ratios of EAA to NEAA were also calculated. Additionally, specific amino acid classifications were determined, including total aromatic amino acids (phenylalanine, tryptophan and tyrosine), taste-related amino acids (asparagine, threonine, serine, glutamic acid, glycine and alanine), umami amino acids (aspartic acid and glutamic acids), and flavor-associated amino acids (valine, isoleucine, leucine, phenylalanine, proline, methionine and arginine) were calculated [38,39]. All analyses were performed in triplicate.

### 2.5. Antioxidant Characteristics of Breast Meat

Breast meat samples (1 g each) were transferred into 15 mL centrifuge tubes, and 5 mL of distilled water was added to each tube. The samples were vortexed for 1 min and then subjected to ultrasonic homogenization for 30 s. Afterward, 3.5 mL of chloroform was added, and the mixture was vortexed and homogenized again. The homogenates were centrifuged at 2100× *g* for 15 min, and the resulting supernatants were collected for assessment of total phenolic content (TPC) and DPPH radical scavenging activity [40].

TPC was measured using the Folin-Ciocalteu assay [41], with gallic acid as the reference standard, as reported by Onbaşılar et al. [42]. Absorbance was read at 760 nm using a spectrophotometer. A calibration curve (y = 6.7765x, R^2^ = 0.9971) was prepared in which y represents absorbance and x denotes gallic acid concentration. TPC values were expressed as mg gallic acid equivalents (GAE) per gram of meat.

DPPH radical scavenging activity was determined using the method reported by Onbaşılar et al. [42]. Absorbance readings were taken at 517 nm, and the percentage inhibition of DPPH radicals was calculated as [1 − (absorbance of the sample/absorbance of the control)] × 100.

For total antioxidant status (TAS) and total oxidant status (TOS) measurements, 1 g of breast meat was placed in 15 mL tubes containing 9 mL of a 140 mM KCl solution. The mixture was homogenized for 30 s using an ultrasonic homogenizer, and then vortexed for 1 min. After centrifugation at 2100× *g* for 10 min, the supernatant was used for analysis [43]. TAS (mmol Trolox equivalents [TE]/kg) and TOS (µmol H_2_O_2_ equivalents/kg) were measured using commercial colorimetric assay kits (Rel Assay Diagnostics, Gaziantep, Turkey; Cat. Nos. RL0017 and RL0024, respectively). The oxidative stress index (OSI) was calculated as (TOS (µmol)/TAS (µmol)) × 100. All measurements were carried out in triplicate.

### 2.6. Statistical Analysis

Data were analysed with IBM SPSS Statistics for Windows, version 23.0 (Armonk, NY: IBM Corp). For variables that met the assumptions of normality, a one-way analysis of variance (ANOVA) was conducted to assess group-related differences. When the ANOVA results indicated significant variation among treatments, Tukey’s multiple comparison test was used to determine specific group differences. A *p*-value below 0.05 was considered indicative of statistical significance.

## 3. Results

Table 4 presents the effects of SW supplementation, administered during different periods and at varying dosages on the appearance and physical properties of broiler breast meat. The initial pH measured 15 min postmortem (pH_15_) did not differ significantly among the groups (*p* > 0.05). However, the ultimate pH (pH_24_) was significantly higher in all SW-supplemented groups than in the control (*p* < 0.001). Despite this increase in ultimate pH, cooking loss and water-holding capacity were not significantly affected by dietary treatments (*p* > 0.05). Regarding meat color, no significant differences were observed in lightness (L*_15_ and L*_24_) or redness (a*_15_ and a*_24_) values at either time point (*p* > 0.05). However, the yellowness (b*_15_ and b*_24_) values were significantly higher in all SW-supplemented groups than in the control group (*p* < 0.001 and *p* < 0.01, respectively).

Table 5 illustrates the effects of supplementation with SW at different periods and dosages on the nutrient composition of broiler breast meat. The results indicate that SW supplementation had no significant impact on dry matter, crude ash, crude fat, or crude protein content (*p* > 0.05).

Table 6 presents the impact of SW supplementation on the amino acid profile of broiler breast meat. Significant differences were observed in alanine, aspartic acid, and glycine levels among the groups (*p* < 0.05). Alanine and aspartic acid levels were lower in some SW-supplemented groups than in the control group, whereas glycine levels were higher in the 1% SW (first 10 days) group. However, other amino acids, including EAA, NEAA, and functional amino acid groups (aromatic, flavor-related, taste-related, and umami AA), were not significantly affected by dietary treatments (*p* > 0.05). Table 7 shows the effects of SW supplementation on the antioxidant capacity of broiler breast meat. No significant differences were observed among the groups for TPC, DPPH radical scavenging activity, TAS, TOS, or OSI (*p* > 0.05).

## 4. Discussion

Shrimp is a seafood in high demand and is primarily processed for its meat, thereby generating more than 50% waste. Globally, a substantial amount of SW is produced each year, creating both an environmental challenge and an opportunity for value-added applications. The analysis of the SW used in this study revealed a high crude protein content of 52.65%, confirming its potential as a protein-rich feed ingredient. The ether extract and crude ash values (9.55% and 18.49%, respectively) indicate a moderate lipid content and a significant mineral fraction, which may contribute to the nutritional quality of diets formulated with SW. Ojewola and Udom [21] reported similar protein and ash values in shrimp meal (53.47% CP and 16.8% CA), although the ether extract content in their study (3.42% EE) was lower than that observed in the present study.

In the present study, the free amino acid profile showed glycine, alanine, and leucine to be the most abundant, whereas total amino acids were dominated by glutamic acid, aspartic acid, leucine, glycine, and alanine. These amino acids are known to play crucial roles in protein synthesis, growth performance, and palatability of feeds for monogastric animals. Glycine, in particular, is involved in collagen formation and overall nitrogen balance, and has been reported to modulate the antioxidant system in shellfish, improving immune function [44]. Free amino acids also contribute to the flavor of foods, including seafood; glutamic acid and aspartic acid can function as flavor-enhancing amino acids, potentially improving feed intake. The presence of EAA such as leucine, lysine, and methionine further underscores the value of SW as a supplementary protein source. Hossain and Shahidi [44] similarly found that glycine was the most abundant free amino acid in shrimp shells, while small amounts of asparagine and glutamine were detected, which is consistent with the present findings.

Meat quality is a key concern for consumers, with pH and color being critical indicators that influence technological properties, shelf life, and overall acceptability. Postmortem pH decline plays a major role in determining meat texture. In the present study, dietary supplementation with 1% or 2% SW across different feeding periods significantly influenced the postmortem pH stabilization in broiler meat. Typically, the ultimate pH_24_ of broiler meat ranges from 5.2 to 6.2, while pale, soft, and exudative (PSE) meat is characterized by a pH below 5.2 [45]. In this study, the pH_24_ values across all treatment groups ranged from 5.64 to 5.77, indicating that inclusion of SW neither caused pre-slaughter stress nor negatively affected rigor mortis.

Meat color, primarily influenced by myoglobin oxidation, is an important quality attribute that reflects both the appearance and the stability of meat. In this study, L* and a* values were not significantly affected by SW supplementation at any time point. However, b* values were significantly higher in all SW groups than in the control, indicating enhanced yellow pigmentation, likely due to dietary carotenoids such as astaxanthin. Although astaxanthin is a red pigment, its accumulation in muscle tissue can intensify yellow tones and improve oxidative stability, thereby enhancing meat color. Similar results were reported by Perenlei et al. [45], who observed increased b* values following supplementation with astaxanthin-rich yeast. Khempaka et al. [28] reported that feeding broilers shrimp meal at levels of 4%, 8%, and 12% increased the redness of thigh meat. They also observed a decrease in breast meat lightness at the 8% and 12% inclusion levels, whereas yellowness and redness were not significantly affected. In a separate study, Hardini and Djunaidi [26] found that supplementing the diet with 5% fermented SW meal did not alter the lightness or redness of broiler breast meat, but significantly increased the yellowness.

The water holding capacity of broiler meat is largely determined by the structural properties of muscle proteins and is a critical technological trait, influencing both meat juiciness and yield during processing [46]. Generally, higher ultimate pH values are associated with improved water-holding capacity and reduced cooking loss due to enhanced protein water-binding capacity [47,48,49]. In the present study, although dietary SW increased ultimate pH, no significant differences in WHC or cooking loss were observed. This aligns with previous reports indicating that minor to moderate pH changes do not necessarily alter WHC or cooking loss, as these traits are also affected by factors such as muscle protein structure and postmortem proteolysis [50]. Bowker and Zhuang [51] highlighted that ultimate pH may have a stronger impact on WHC than does myofibrillar protein denaturation in processed broiler breast fillets. In another study, the inclusion of fermented SW meal at higher levels (7.5–12.5%) reduced cooking loss, while WHC decreased only at those levels, with no significant change in pH [26].

The chemical composition of meat is an important indicator of broiler meat quality, nutritional value, and potential health benefits. Factors such as diet, age, rearing environment, and the specific muscle cut can influence these characteristics [52,53]. In this study, dietary supplementation with SW at different levels and time points did not significantly alter the nutrient composition of breast meat. In contrast, previous research has shown that higher inclusion levels of fermented SW meal (5–12.5%) reduce fat content, whereas protein and ash levels remain unaffected [26]. Overall, the nutrient values observed in the present study are consistent with values reported for broilers under various feeding regimes and management conditions [18,53,54].

Amino acid quantities and proportions are key determinants of protein quality because they participate in numerous metabolic and physiological processes [55]. In the present study, alanine, phenylalanine, glycine, lysine, isoleucine, leucine, methionine, proline, tyrosine, and valine were the predominant amino acids in breast meat across all groups. EAA cannot be synthesized endogenously and, therefore, must be obtained from the diet. Adequate EAA intake contributes to efficient protein utilization and overall metabolic performance [56]. NEAA, such as alanine, glycine, and aspartic acid, though synthesized internally, play crucial roles in nitrogen balance, intermediary metabolism, and the sensory properties of meat. Free amino acids influence flavor: glutamic and aspartic acids are associated with umami, whereas alanine, glycine, serine, proline, threonine, and asparagine contribute to sweetness [57]. Consequently, the concentrations of essential, sweet-tasting, and umami-related amino acids in muscle tissue are important determinants of nutritional quality and palatability [57,58].

In this study, alanine levels were significantly lower in the group receiving 1% SW during the first 10 days compared with the control group. Because alanine is a glucogenic amino acid involved in energy metabolism, reduced alanine levels may indicate shifts in protein turnover or energy utilization in response to the inclusion of SW. Given its contribution to meat sweetness, such changes could also influence sensory attributes. Aspartic acid was significantly reduced in most SW groups, except in the 1% SW group throughout the trial. Because aspartic acid is a key component of protein biosynthesis and nitrogen metabolism and a precursor to EAA such as lysine, threonine, and methionine, alterations in aspartic acid may reflect modifications in metabolic pathways linked to growth and muscle development. Glycine, which increased significantly in the 1% SW group during the first 10 days, is involved in collagen synthesis, meat tenderness, detoxification pathways, and immune function [59]. Its elevation may indicate enhanced connective tissue synthesis, potentially affecting meat texture. These responses align with previous evidence suggesting that dietary interventions can modify amino acid profiles, with subsequent effects on meat flavor and consumer acceptability [57,60]. The observed changes in alanine, aspartic acid, and glycine levels may be related to the nutritional characteristics of SW, particularly its chitin content and amino acid profile. Chitin, a nitrogen-containing polysaccharide, may influence nitrogen metabolism by reducing protein digestibility and amino acid bioavailability. Although poultry produce acidic chitinase in the proventriculus, the digestion of chitin remains limited, which may affect nitrogen utilization and amino acid deposition [61]. Supporting this, studies using chitosan in broilers have shown increased nitrogen retention, indicating that chitin derivatives can modulate nitrogen metabolism [62]. Nevertheless, since most amino acids were unaffected, SW supplementation at low inclusion levels appears not to markedly disrupt the overall amino acid composition of broiler breast meat.

Overall, ratios of EAA to NEAA and levels of aromatic, flavor-related, sweet-tasting, and umami-associated amino acids remained largely stable. This suggests that the inclusion levels tested do not compromise protein quality. However, the specific alterations observed in alanine, aspartic acid, and glycine indicate subtle shifts in nitrogen partitioning and protein metabolism, highlighting the need for further research into the digestibility and enzymatic degradation of chitin-containing feed ingredients and their long-term impacts on amino acid bioavailability and meat quality.

Feeding broilers with antioxidant-rich ingredients is known to enhance meat quality, as the antioxidant capacity of breast meat reflects both oxidative stability and overall health. Assessing antioxidant status through multiple analytical methods provides broader insight, indicating that compounds other than phenolics can contribute to antioxidant activity in animal tissues [63]. SW contains astaxanthin, a carotenoid with strong antioxidative properties [64]. However, in the present study, SW supplementation did not significantly influence TPC, DPPH radical scavenging activity, TAS, TOS, or OSI values. The absence of measurable effects may be attributed to the relatively low inclusion level of SW. Thus, future studies employing higher or graded doses are warranted to better elucidate its impact on antioxidant capacity, meat quality, and color. Supporting this notion, Hosseindoust et al. [65] demonstrated that dietary astaxanthin supplementation (40 and 80 mg/kg) improved 3-ethylbenzothiazoline-6-sulfonate (ABTS) and DPPH radical scavenging capacity and enhanced antioxidant capacity in the leg muscle, indicating reduced oxidative tissue damage. This evidence suggests that higher levels of astaxanthin, such as those potentially provided by increased inclusion of SW, may be required to yield detectable improvements in oxidative stability.

## 5. Conclusions

The global population continues to grow rapidly, increasing the demand for both food and energy. Poultry meat, due to its high production efficiency, plays an important role in meeting this rising food demand. At the same time, feed costs in poultry production are escalating, promoting interest in alternative feed resources, particularly those derived from agro-industrial waste streams. The inclusion of such by-products can help reduce feed expenses and support environmental sustainability, provided that meat quality is not compromised. In the present study, supplementing broiler diets with shrimp waste did not adversely affect meat quality, indicating its potential as a sustainable feed ingredient. Although slight alterations were detected in alanine, aspartic acid, and glycine concentrations, the overall amino acid profile remained largely unchanged, suggesting that shrimp waste does not diminish the nutritional value of broiler breast meat. These findings highlight the feasibility of integrating shrimp waste into broiler diets as a strategy to lower feed costs and promote environmental sustainability, while maintaining meat quality and composition.

## Figures and Tables

**Table 1:** Experimental groups.

Group	0–11 days	11–42 days	Description
1 (Control)	-	-	No SW supplementation
2	1% SW	-	1% SW supplementation only during the first 10 days
3	1% SW	1% SW	1% SW supplementation throughout the entire trial
4	2% SW	-	2% SW supplementation only during the first 10 days
5	2% SW	2% SW	2% SW supplementation throughout the entire trial

SW: Shrimp waste.

**Table 2:** Amino acid composition of shrimp waste.

Amino Acids	Free Amino Acids mg/kg Dry Matter	Total Amino Acids mg/kg Dry Matter
Phenylalanine	1047	16,059
Leucine	2962	28,653
Isoleucine	1177	13,060
Tryptophan	317	317
Methionine	174	5809
Valine	935	16,660
Tyrosine	168	5489
Proline	1380	11,461
Glutamic acid	1803	59,280
Threonine	619	18,591
Alanine	3188	20,013
Aspartic acid	304	38,022
Glutamine	162	162
Asparagine	348	348
Serin	541	16,724
Gamma amino butyric acid	28	262
Glycine	3658	23,466
Histidine	161	9611
Ornithine	54	602
Lysine	1244	18,813
Arginine	1739	16,770
Total	22,009	319,345

**Table 3:** Ingredients and composition of diets.

Ingredients	Groups for 0–11 Days			Groups for 11–25 Days			Groups for 25–42 Days		
	1	2, 3	4, 5	1, 2, 4	3	5	1, 2, 4	3	5
Maize	46.310	47.275	48.160	48.655	49.487	50.420	55.635	56.420	57.240
Fullfat soya	23.220	21.360	20.070	23.395	22.650	21.107	24.255	23.800	23.220
Soybean meal	25.170	25.260	24.890	21.750	21.020	20.851	13.890	12.960	12.110
Dry shrimp waste	0	1.000	2.000	0	1.000	2.000	0	1.000	2.000
Soy oil	1.040	1.130	1.130	2.300	2.190	2.220	2.930	2.780	2.640
Limestone	1.500	1.250	1.040	1.400	1.170	0.939	1.140	0.910	0.680
Monocalcium phosphate	1.500	1.460	1.420	1.400	1.360	1.322	1.200	1.160	1.120
DL-Methionine	0.250	0.250	0.250	0.200	0.199	0.198	0.160	0.160	0.160
L-Lysine	0.180	0.185	0.210	0.100	0.119	0.138	0.080	0.100	0.120
L-Threonine	0.100	0.100	0.100	0.070	0.075	0.075	0.030	0.030	0.030
Choline	0.080	0.080	0.080	0.080	0.080	0.080	0.080	0.080	0.080
Sodium bicarbonate	0.150	0.150	0.150	0.150	0.150	0.150	0.150	0.150	0.150
Salt	0.250	0.250	0.250	0.250	0.250	0.250	0.250	0.250	0.250
Vitamin premix ^1^	0.100	0.100	0.100	0.100	0.100	0.100	0.100	0.100	0.100
Mineral premix ^2^	0.100	0.100	0.100	0.100	0.100	0.100	0.100	0.100	0.100
Anticoccidial ^3^	0.050	0.050	0.050	0.050	0.050	0.050	0	0	0
Analyzed values									
Dry matter, %	89.72	90.03	89.82	89.31	89.32	89.82	89.03	89.78	89.71
Crude protein, %	23.05	23.07	23.08	21.53	21.51	21.51	19.58	19.51	19,56
ME ^4^, kcal/kg	3010	3007	3014	3112	3114	3115	3205	3201	3202

^1^: Each kg provided 11,000,000 IU of vitamin A, 3,500,000 IU of vitamin D3, 100 g of vitamin E, 3 g of vitamin K3, 3 g of vitamin B1, 6 g of vitamin B2, 15 g of calcium D-pantothenate, 1 g of vitamin B6, 20 mg of vitamin B12, 35 g of niacin, 1.5 g of folic acid, and 200 mg of D-biotin, ^2^: Each kilogram supplied 30 g of copper, 120 g of manganese, 110 g of zinc, 2 g of iodine, 300 mg of selenium, and 50 g of iron, ^3^: Salinomycin, ^4^: Metabolizable energy, calculated.

**Table 4:** Effects of dietary shrimp waste inclusion at different levels and feeding phases on the appearance and physical characteristics of broiler breast meat.

Parameters	Control	1% SW During the First 10 Days	1% SW Throughout the Entire Trial	2% SW During the First 10 Days	2% SW Throughout the Entire Trial	*p*-Value
pH_15_	6.48 ± 0.08	6.43 ± 0.09	6.32 ± 0.11	6.41 ± 0.10	6.16 ± 0.14	0.271
pH_24_	5.64 ± 0.03 b	5.77 ± 0.02 a	5.74 ± 0.01 a	5.76 ± 0.02 a	5.75 ± 0.02 a	<0.001
L*_15_	45.52 ± 0.76	44.65 ± 0.61	46.35 ± 0.71	46.28 ± 0.56	46.84 ± 0.94	0.268
a*_15_	2.91 ± 0.40	3.45 ± 0.32	4.09 ± 0.22	3.23 ± 0.21	3.35 ± 0.49	0.204
b*_15_	6.46 ± 0.32 b	8.90 ± 0.44 a	10.95 ± 0.56 a	9.43 ± 0.76 a	9.44 ± 0.50 a	<0.001
L*_24_	53.92 ± 1.00	51.42 ± 0.89	53.16 ± 0.96	51.68 ± 0.85	52.16 ± 0.59	0.243
a*_24_	1.73 ± 0.24	1.71 ± 0.12	2.14 ± 0.25	2.26 ± 0.30	1.83 ± 0.26	0.383
b*_24_	9.20 ± 0.40 b	11.77 ± 0.79 a	12.56 ± 0.53 a	11.55 ± 0.29 a	11.76 ± 0.49 a	0.001
Cooking loss (%)	30.86 ± 0.78	30.45 ± 0.48	32.10 ± 0.87	32.43 ± 0.82	30.88 ± 0.82	0.303
Water holding capacity (%)	18.99 ± 1.81	17.80 ± 1.42	19.67 ± 1.07	19.53 ± 0.76	18.41 ± 1.35	0.844

Mean ± Standard error of mean. SW: shrimp waste, pH_15_: pH value at 15 min, pH_24_: pH value at 24 h, L*_15_: lightness value at 15 min, a*_15_: redness value at 15 min, b*_15_: yellowness value at 15 min, L*_24_: lightness value at 24 h, a*_24_: redness value at 24 h, b*_24_: yellowness value at 24 h, a, b; difference between values with different letters on the same line is statistically significant (*p* < 0.05).

**Table 5:** Effects of dietary shrimp waste inclusion at different levels and feeding phases on the nutrient composition of broiler breast meat.

Parameters	Control	1% SW During the First 10 Days	1% SW Throughout the Entire Trial	2% SW During the First 10 Days	2% SW Throughout the Entire Trial	*p*-Value
Dry matter, %	24.27 ± 0.19	24.23 ± 0.33	24.35 ± 0.27	24.28 ± 0.34	24.35 ± 0.31	0.998
Crude ash, %	1.16 ± 0.02	1.16 ± 0.01	1.14 ± 0.01	1.15 ± 0.02	1.14 ± 0.03	0.913
Ether extract, %	0.88 ± 0.08	0.89 ± 0.13	1.02 ± 0.12	0.97 ± 0.10	1.09 ± 0.16	0.699
Crude protein, %	22.24 ± 0.19	22.19 ± 0.36	22.20 ± 0.28	22.16 ± 0.31	22.11 ± 0.24	0.999

Mean ± Standard error of mean, SW: shrimp waste.

**Table 6:** Effects of dietary shrimp waste inclusion at different levels and feeding phases on amino acid profiles (% total amino acids) of broiler breast meat.

Parameters	Control	1% SW During the First 10 Days	1% SW Throughout the Entire Trial	2% SW During the First 10 Days	2% SW Throughout the Entire Trial	*p*-Value
Alanine	9.34 ± 0.30 a	7.48 ± 0.13 c	8.76 ± 0.18 ab	8.24 ± 0.31 bc	8.50 ± 0.43 ab	0.002
Arginine	3.23 ± 0.14	3.47 ± 0.17	3.18 ± 0.11	3.25 ± 0.13	3.10 ± 0.16	0.455
Asparagine	0.61 ± 0.07	0.83 ± 0.09	0.52 ± 0.10	0.57 ± 0.03	0.60 ± 0.04	0.054
Aspartic acid	3.17 ± 0.22 a	2.37 ± 0.13 b	2.79 ± 0.18 ab	2.49 ± 0.18 b	2.47 ± 0.16 b	0.020
Phenylalanine	8.35 ± 0.18	8.09 ± 0.35	8.78 ± 0.19	8.37 ± 0.16	8.52 ± 0.20	0.324
Glycine	6.42 ± 0.36 b	8.66 ± 0.89 a	7.67 ± 0.76 ab	6.14 ± 0.47 b	6.27 ± 0.64 b	0.042
Glutamic acid	3.21 ± 0.14	3.02 ± 0.19	3.19 ± 0.18	3.29 ± 0.13	3.00 ± 0.13	0.610
Glutamine	3.25 ± 0.17	2.93 ± 0.17	3.17 ± 0.13	2.99 ± 0.15	2.83 ± 0.10	0.258
Histidine	3.21 ± 0.13	3.47 ± 0.18	3.13 ± 0.15	3.43 ± 0.09	3.39 ± 0.13	0.367
Lysine	5.25 ± 0.24	5.34 ± 0.28	5.27 ± 0.29	5.69 ± 0.24	5.46 ± 0.23	0.744
Leucine and Isoleucine	11.07 ± 0.34	10.32 ± 0.45	11.23 ± 0.30	11.08 ± 0.29	11.55 ± 0.24	0.146
Methionine	6.02 ± 0.34	6.17 ± 0.37	6.29 ± 0.29	6.81 ± 0.31	6.81 ± 0.33	0.303
Proline	7.64 ± 0.53	7.71 ± 0.35	6.54 ± 0.57	7.17 ± 0.47	7.17 ± 0.41	0.430
Serine	4.02 ± 0.24	4.27 ± 0.29	4.43 ± 0.21	3.83 ± 0.35	4.09 ± 0.31	0.624
Cysteine	0.30 ± 0.07	0.29 ± 0.06	0.31 ± 0.03	0.41 ± 0.11	0.33 ± 0.05	0.704
Tyrosine	10.50 ± 0.39	10.97 ± 0.40	10.76 ± 0.42	10.57 ± 0.32	10.77 ± 0.41	0.920
Threonine	2.51 ± 0.16	2.45 ± 0.11	2.38 ± 0.07	2.39 ± 0.18	2.33 ± 0.11	0.891
Tryptophan	1.64 ± 0.03	1.62 ± 0.06	1.61 ± 0.05	1.55 ± 0.07	1.53 ± 0.06	0.534
Valine	10.27 ± 0.50	10.54 ± 0.49	10.00 ± 0.44	11.72 ± 0.44	11.30 ± 0.58	0.100
∑EAA	62.05 ± 1.24	62.46 ± 1.34	62.64 ± 0.63	64.86 ± 1.21	64.74 ± 1.40	0.306
∑NEAA	37.95 ± 1.24	37.54 ± 1.34	37.37 ± 0.63	35.14 ± 1.21	35.26 ± 1.40	0.306
∑EAA/∑NEAA	1.65 ± 0.09	1.69 ± 0.10	1.68 ± 0.05	1.87 ± 0.10	1.86 ± 0.11	0.271
Aromatic AA	20.50 ± 0.48	20.69 ± 0.58	21.15 ± 0.46	20.48 ± 0.32	20.81 ± 0.50	0.853
Flavour-related AA	46.58 ± 0.82	46.31 ± 1.11	46.02 ± 0.96	48.41 ± 0.67	48.44 ± 1.15	0.224
Tasty AA	26.10 ± 0.65	26.70 ± 0.99	26.95 ± 0.66	24.47 ± 0.67	24.79 ± 1.29	0.203
Umami AA	6.38 ± 0.28	5.38 ± 0.25	5.97 ± 0.31	5.78 ± 0.26	5.47 ± 0.17	0.066

Mean ± Standard error of mean, SW: shrimp waste, AA: amino acids, EAA: Essential amino acids, NEAA: nonessential amino acids, a, b, c; difference between values with different letters on the same line is statistically significant (*p* < 0.05).

**Table 7:** Effects of dietary shrimp waste inclusion at different levels and feeding phases on antioxidant capacity of broiler breast meat.

Parameters	Control	1% SW During the First 10 Days	1% SW Throughout the Entire Trial	2% SW During the First 10 Days	2% SW Throughout the Entire Trial	*p*-Value
TPC, mg GAE/g	0.94 ± 0.04	0.98 ± 0.07	0.95 ± 0.06	0.93 ± 0.03	0.95 ± 0.04	0.960
DPPH RSA, %	74.89 ± 2.27	75.77 ± 2.60	70.91 ± 3.26	68.48 ± 2.23	66.83 ± 2.91	0.104
TAS, mmol TE/kg	10.02 ± 0.39	10.38 ± 0.65	9.26 ± 0.24	9.94 ± 0.37	10.19 ± 0.54	0.496
TOS, µmol H_2_O_2_ E/kg	14.19 ± 0.76	12.86 ± 1.59	14.92 ± 1.33	11.28 ± 1.66	12.37 ± 0.74	0.300
OSI	0.14 ± 0.01	0.13 ± 0.03	0.16 ± 0.01	0.12 ± 0.02	0.12 ± 0.01	0.417

Mean ± Standard error of mean, SW: shrimp waste, TPC: total phenolic content, GAE: gallic acid equivalent, DPPH RSA: 2,2-diphenyl-1-picrylhydrazyl radical scavenging activity, TAS: total antioxidant status, TE: Trolox Equivalent, TOS: total oxidant status, E: equivalent, OSI: oxidative stress index.

## Data Availability

The original contributions presented in this study are included in the article. Further inquiries can be directed to the corresponding authors.

## Abbreviations

The following abbreviation is used in this manuscript:

AA	Amino acids
CA	Crude ash
CF	Crude fibre
CP	Crude protein
DM	Dry matter
EAA	Essential amino acids
EE	Ether extract
GAE	Gallic acid equivalent
NEAA	Non-essential amino acids
OSI	Oxidative stress index
SW	Shrimp waste
TAS	Total antioxidant status
TE	Trolox equivalent
TOS	Total oxidant status
TPC	Total phenolic content
WHC	Water holding capacity
